# Comparing the Effectiveness and Image Quality of Musculoskeletal Ultrasound of First-Year Medical Students After Training by Student Tutors Versus Ultrasound Instructors: A Pilot Study

**DOI:** 10.7759/cureus.26890

**Published:** 2022-07-15

**Authors:** Jeffrey J Li, Jane J Kim, Corey Young, Fauzia Nausheen

**Affiliations:** 1 Education, California University of Science and Medicine, Colton, USA

**Keywords:** ultrasound (u/s), curriculum, student tutor, peer teaching, musculoskeletal

## Abstract

Background

Ultrasound is a vital part in many medical schools’ curriculum. Although there is strong support for the use of student tutors (STs), there is a lack in gauging their effectiveness with more difficult organ systems such as the musculoskeletal (MSK) system. We aim to determine the effectiveness of using STs versus expert ultrasound instructors (UIs) when teaching MSK ultrasound.

Methodology

Medical students were recruited to participate in an MSK workshop to identify superficial volar arm structures (radial nerve, radial artery, median nerve, ulnar artery, ulnar nerve) using Butterfly iQ. In total, 14 participants were taught by STs and 10 participants were taught by UIs. Participants imaged the five structures and answered surveys gauging their confidence via five-point Likert scales.

Results

There was no significant difference in confidence or identification accuracy for all five structures between the two groups. However, there was a significant difference in confidence in the understanding of basic ultrasound concepts in favor of the UI group (p < 0.05). A greater number of students were more confident in identifying all five structures when taught by STs, but more students correctly identified the structures when taught by UIs.

Conclusions

The results on confidence could be due to positive peer perception. Both groups scored relatively high in their identification accuracies, promoting the successful use of STs. The successful use of STs in teaching MSK ultrasound opens the possibility to developing peer-led ultrasound curriculum on more complex ultrasound topics in the future.

## Introduction

The portability, low cost, and easy accessibility of handheld ultrasound probes have quickly made ultrasound the first choice diagnostic tool for exploring many musculoskeletal (MSK) conditions [1]. Despite the utility and widespread use of ultrasound for MSK conditions, MSK ultrasound can be more difficult to master than other forms of ultrasound due to high image variability caused by movement, frequently encountered artifacts when scanning uneven surfaces, and the general complexities of how MSK pathophysiology can change ultrasound images [2]. In comparison with other ultrasound exams, such as right upper quadrant views, FAST (Focused Assessment with Sonography in Trauma) exam views, and cardiac views, MSK joint ultrasound has still been shown to be more difficult for many preclinical medical students to perform [3]. In addition, when ranking which form of point-of-care ultrasound was the most unfamiliar among 854 Canadian family medicine residents, MSK ultrasound ranked the highest [4]. Despite the difficulty and lack of exposure to MSK ultrasound, it is rapidly becoming integrated into medical education for residents in MSK-related specialties, such as physical medicine and rehabilitation and orthopedic surgery, due to its ability to diagnose a high variety of common MSK conditions [5,6]. Point-of-care ultrasound in general has become widely adopted into medical school preclinical curricula as well [7]. The scarcity of ultrasound faculty as well as the need for small group sessions to allow sufficient hands-on ultrasound practice time have been barriers in medical school ultrasound education. However, studies have shown the efficacy of using student tutors (STs) as a way to combat the lack of faculty resources in teaching ultrasound at the preclinical level [8]. Furthermore, studies supported that trained STs can teach their peers effectively [9].

We created a series of student-led ultrasound workshops that focused on exposing preclinical students to the different organ systems and uses of point-of-care ultrasound. We paralleled the anatomy of the ultrasound workshop with the students’ school curriculum when possible to synergistically build their system-based knowledge. Previous literature demonstrates that preclinical ultrasound training can help improve student proficiency in anatomy and physiology [10]. The broad scope of ultrasound to improve physician diagnostic ability while enhancing medical understanding encourages the implementation of an ultrasound curriculum to improve both future patient outcomes and preclinical student knowledge.

This pilot study’s objective was to determine whether STs are still effective versus ultrasound instructors (UIs) when teaching an organ system that has more complexities such as MSK ultrasound. We also aimed to determine whether students’ confidence correlated with their accuracy of MSK ultrasound imaging.

## Materials and methods

Informed written consent was obtained from all participants prior to their participation. This study was approved by the local Institutional Review Board (protocol number: HS-2022-12).

We sent out emails to Year 1 medical students for participation in an MSK ultrasound workshop with the goal of identifying superficial structures of the volar arm. From lateral to medial, these structures included the radial nerve, radial artery, median nerve, ulnar artery, and ulnar nerve. For teaching and congruity purposes, the distal half of the volar arm was used for the identification of these five structures. For assessment purposes, all ultrasound images were taken and saved in the short axis. In total, 40 students participated in the workshop. Of those 40, we screened 24 students as ultrasound-naive, and they were randomized to be taught by either STs or UIs. STs were medical students with less than one year of ultrasound experience who were trained and approved by expert ultrasound faculty to teach their peers. UIs were ultrasound-trained physicians or senior medical students with more than five years of ultrasound experience. After the teaching session, participants took images and labeled the aforementioned arteries and nerves on their own via Butterfly iQ ultrasound probes. Independent, blinded, faculty-trained reviewers analyzed these images post-workshop for accuracy and quality. Additionally, participants were given an anonymous survey of five-point Likert scale statements that consisted of the answers “strongly disagree” (1), “disagree” (2), “neutral” (3), “agree” (4), and “strongly agree” (5), which they were able to fill out on their own time. These questions gauged the students’ confidence in identifying the radial nerve, radial artery, median nerve, ulnar artery, and ulnar nerve. In addition to rating confidence in identification, students were given the following statements where they rated their confidence on the same five-point Likert scale:

“I feel comfortable explaining basic ultrasound concepts (e.g. echogenicity, how to read images, transverse/longitudinal views).”

“I am overall confident in identifying volar arm nerves and arteries using ultrasound.”

“The amount of instruction provided prior to working with the ultrasound machine was adequate.”

“I feel confident in ultrasound after my ultrasound training session.”

We grouped the responses to the four statements above with “1-2” as “disagree,” “3” as “neutral,” and “4-5” as “agree.”

We used Wilcoxon rank tests to assess the independence of both groups in their survey responses. We implemented Chi-squared analysis with Yates correction to test for statistical differences in identification accuracy between both groups. We also used Goodman and Kruskal’s gamma to identify the association between the students’ perceived confidence in identifying each structure (radial nerve, radial artery, median nerve, ulnar artery, ulnar nerve) and their actual accuracies in identifying the corresponding structures. Each participant then received a summative score from 0 to 5, depending on how many of the five structures they were able to identify (0 = no structures identified, 5 = all five structures identified). We used Goodman and Kruskal’s gamma again to show the association between the students’ perceived overall confidence (“I am overall confident in identifying volar arm nerves and arteries using ultrasound” and “I feel confident in ultrasound after my ultrasound training session”) and their ability to identify structures using their summative scores. This analysis revealed correlations between students’ overall confidence and their overall ability to identify volar arm structures.

## Results

In total, 14 students were taught by STs and 10 students were taught by UIs. Surprisingly, the number of “agree” and “strongly agree” responses for self-reported confidence was higher in the ST group compared with the UI group for all five structures. According to Table 1 and Table 2, 57% of ST-taught students agreed they were confident in identifying the radial nerve compared with 40% of UI-taught students. Overall, 93% of ST-taught students were confident in identifying the radial artery compared with 70% of UI-taught students. Overall, 72% of ST-taught students were confident in identifying the median nerve while 50% of UI-taught students were confident in doing so. Moreover, 71% of ST-taught students agreed they were confident in identifying the ulnar artery compared to 60% of UI-taught students. Finally, 64% of ST-taught students were confident in identifying the ulnar nerve compared with 50% of UI-taught students. The structure that most students agreed on feeling confident in identifying was the radial artery for both groups. The structure that most students did not “agree” to be confident in identifying was the ulnar nerve. Despite the consistently higher number of self-reported confidence levels of ST-taught students, Wilcoxon rank tests revealed that they did not reach statistical significance (p > 0.05).

**Table 1:** Student confidence and accuracy of identifying volar arm structures using ultrasound when taught by STs or UIs. ST = student tutor; UI = ultrasound instructor

Structures	Teacher	Student confidence in identification					
		Strongly disagree	Disagree	Neutral	Agree	Strongly agree	Median (range)
I am confident in identifying the radial nerve on ultrasound	ST	0	1	5	7	1	4 (2-4)
		(0.0%)	(7.1%)	(35.7%)	(50.0%)	(7.1%)	
	UI	0	1	5	2	2	3 (2-5)
		(0.0%)	(10.0%)	(50.0%)	(20.0%)	(20.0%)	
I am confident in identifying the radial artery on ultrasound	ST	0	0	1	7	6	4 (3-5)
		(0.0%)	(0.0%)	(7.1%)	(50.0%)	(42.9%)	
	UI	0	2	1	1	6	5 (2-5)
		(0.0%)	(20.0%)	(10.0%)	(10.0%)	(60.0%)	
I am confident in identifying the median nerve on ultrasound	ST	0	1	3	6	4	4 (2-5)
		(0.0%)	(7.1%)	(21.4%)	(42.9%)	(28.6%)	
	UI	0	3	2	2	3	3.5 (2-5)
		(0.0%)	(30.0%)	(20.0%)	(20.0%)	(30.0%)	
I am confident in identifying the ulnar artery on ultrasound	ST	0	0	4	5	5	4 (3-5)
		(0.0%)	(0.0%)	(28.6%)	(35.7%)	(35.7%)	
	UI	0	1	3	1	5	4.5 (2-5)
		(0.0%)	(10.0%)	(30.0%)	(10.0%)	(50.0%)	
I am confident in identifying the ulnar nerve on ultrasound	ST	0	1	4	6	3	4 (2-5)
		(0.0%)	(7.1 %)	(28.6%)	(42.9%)	(21.4%)	
	UI	1	1	3	2	3	3.5 (1-5)
		(10.0%)	(10.0%)	(30.0%)	(20.0%)	(30.0%)	

**Table 2:** Comparison of accuracy, using Chi-squared with Yates correction, and confidence levels, using Wilcoxon rank tests, of ultrasound identification when taught by STs or UIs. ST = student tutor; UI = ultrasound instructor; df = degrees of freedom

Structures	Teacher	Could identify	Could not identify	X^2^ (df = 1)	P-value	Median confidence (range)	U	Z	P-value
Radial nerve	ST	11 (78.6%)	3 (21.4%)	0.034	0.853	4 (2-4)	76	0.345	0.731
	UI	9 (90.0%)	1 (10.0%)			3 (2-5)			
Radial artery	ST	13 (92.9%)	1 (7.1%)	0.745	0.388	4 (3-5)	69	-0.032	0.975
	UI	10 (100%)	0 (0%)			5 (2-5)			
Median nerve	ST	13 (92.9%)	1 (7.1%)	0.062	0.803	4 (2-5)	83.5	0.791	0.429
	UI	9 (90.0%)	1 (10.0%)			3.5 (2-5)			
Ulnar artery	ST	13 (92.9%)	1 (7.1%)	0.745	0.388	4 (3-5)	70	-0.031	0.975
	UI	10 (100%)	0 (0%)			4.5 (2-5)			
Ulnar nerve	ST	12 (85.7%)	2 (14.3%)	0.137	0.711	4 (2-5)	77	0.396	0.692
	UI	8 (80.0%)	2 (20.0%)			3.5 (1-5)			

Conversely, UI-taught students scored higher in more categories when it came to correctly identifying volar arm structures. As shown in Table 2, 100% of UI-taught students correctly identified the radial and ulnar arteries, and 90% correctly identified the median and radial nerves. However, 80% of students correctly identified the ulnar nerve. Comparatively, 93% of ST-taught students correctly identified the radial and ulnar arteries and median nerve. However, only 79% correctly identified the radial nerve, and 86% correctly identified the ulnar nerve. UI-taught students scored higher in identification of the radial nerve, radial artery, and ulnar artery compared to ST-taught students. The chi-squared test with Yates correction demonstrated that these two populations had no statistically significant differences in identification accuracy for all five structures (p > 0.5).

**Figure 1:**
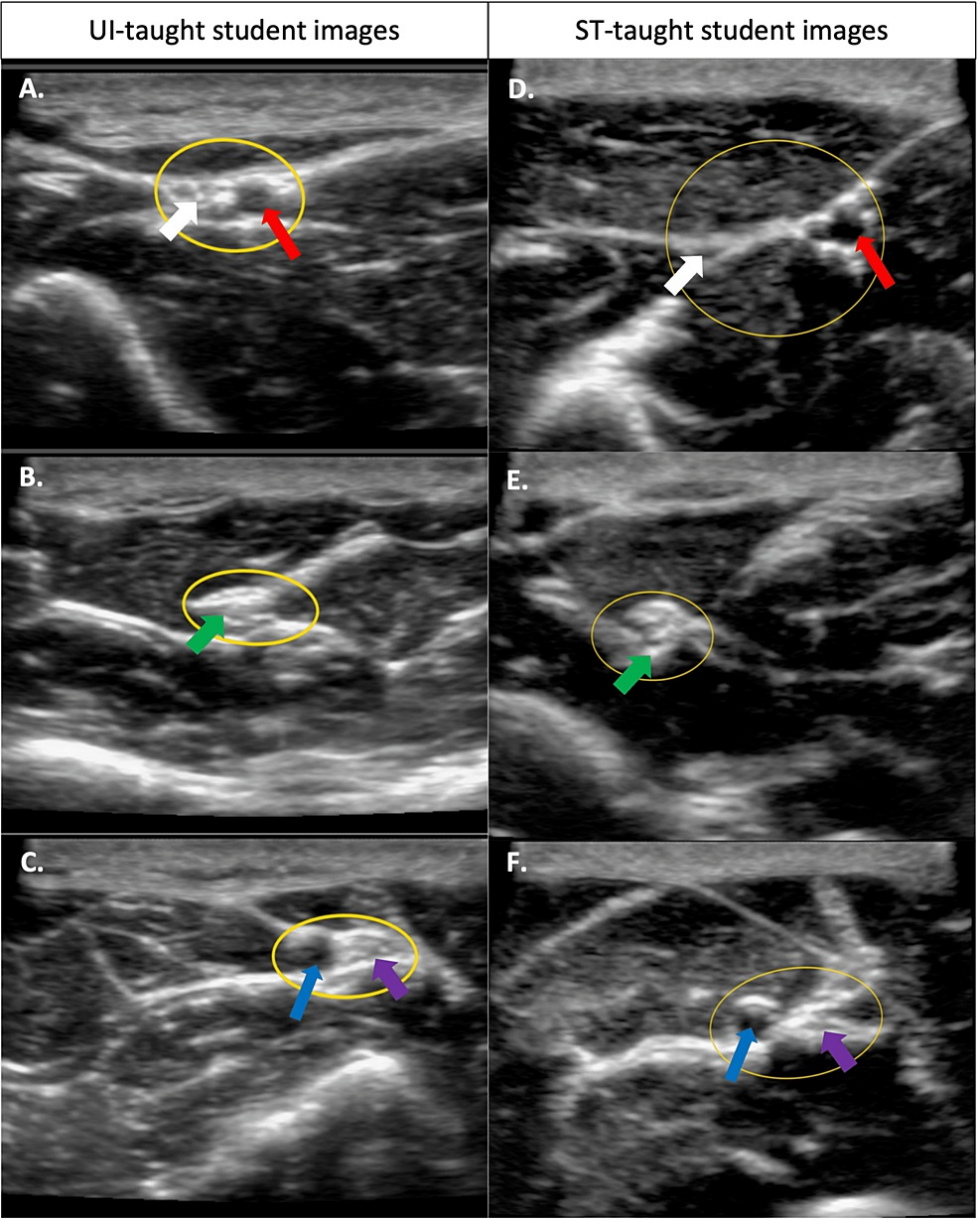
Comparison of ultrasound image quality taken by students taught by UIs or STs. A, B, and C were imaged by the UI group, and D, E, and F were imaged by the ST group. Yellow circle (part of the original image) = made by students to identify nerves and arteries; white arrow = radial nerve; red arrow = radial artery; green arrow = median nerve; blue arrow = ulnar artery; purple arrow = ulnar nerve; ST = student tutor; UI = ultrasound instructor

As shown in Table 3, 100% agreed that instruction prior to the usage of the ultrasound probe was sufficient, only 43% of students taught by STs agreed that they felt comfortable explaining basic ultrasound concepts, 57% agreed that they were overall confident in identifying volar arm nerves and arteries using ultrasound, and 64% agreed that they felt comfortable using ultrasound in general after the workshop. In contrast, 90% felt instruction to use the ultrasound probe was sufficient, 90% of UI-taught students felt comfortable explaining basic ultrasound concepts, only 40% felt overall confident in identifying volar arm nerves and arteries, and 80% felt comfortable using ultrasound in general after the workshop. There were no significant differences in how the two groups responded to these statements with the exception of “I feel comfortable explaining basic ultrasound concepts,” where UI-taught students (median = 5, n = 10) responded more favorably compared to ST-taught students (median = 3, n = 14, p < 0.05). Figure 1 shows the comparison of the average quality of the images taken by the students taught by STs and UIs. There was no significant difference in the image quality with relevance to time gain compensation, depth, and precision.

**Table 3:** Comparison, using Wilcoxon rank tests, of confidence levels in overall ultrasound skills between the ST and UI group. ST = student tutor; UI = ultrasound instructor; * = p < 0.05

Statement	Teacher	Count	Disagree	Neutral	Agree	Median (range)	U	Z	P-Value
I feel comfortable explaining basic ultrasound concepts	ST	14	0%	57.1%	42.9%	3 (3-5)	29	-2.505	0.012*
	UI	10	10.0%	0%	90.0%	5 (2-5)			
The amount of instruction provided prior to working with the ultrasound machine was adequate	ST	14	0%	0%	100.0%	5 (4-5)	73	0.184	0.854
	UI	10	10.0%	0%	90.0%	5 (2-5)			
I am overall confident in identifying volar arm nerves and arteries using ultrasound	ST	14	7.1%	35.7%	57.1%	4 (2-4)	70	-0.031	0.975
	UI	10	10.0%	50.0%	40.0%	3 (2-5)			
I feel confident in ultrasound after my ultrasound training session	ST	14	0%	35.7%	64.3%	4 (3-4)	52.5	-1.157	0.248
	UI	10	10.0%	10.0%	80.0%	4 (2-5)			

As shown in Table 4, there were no statistically significant correlations between the students’ confidence and their abilities to identify individual volar arm structures for both groups (p > 0.05). Furthermore, there were also no significant correlations of the statements “I am overall confident in identifying volar arm nerves and arteries using ultrasound” or “I feel confident in ultrasound after my ultrasound training session” compared to the summative performance scores of the students, with the exception of a significant positive correlation between the ST group’s agreement with the statement “I am overall confident in identifying volar arm nerves and arteries using ultrasound” and their overall identification accuracy (γ = 0.778, p < 0.01).

**Table 4:** Goodman Kruskal gamma scores for correlation between perceived confidence in the identification of radial nerve, radial artery, median nerve, ulnar artery, and ulnar nerve, and actual accuracy in identification. In addition, identifies any correlation of overall identification accuracy with overall confidence in identifying nerves/arteries or general ultrasound. ST = student tutor; UI = ultrasound instructor; * = p < 0.05

Structures	Teacher	γ	P-value
Radial nerve	ST	0.429	0.375
	UI	0.6	0.352
Radial artery	ST	1	0.261
	UI	N/A	N/A
Median nerve	ST	-1	0.268
	UI	1	0.246
Ulnar artery	ST	0.111	0.748
	UI	N/A	N/A
Ulnar nerve	ST	1	0.074
	UI	0.077	0.849
I am overall confident in identifying volar arm nerves and arteries using ultrasound	ST	0.778	0.002*
	UI	0.6	0.153
I feel confident in ultrasound after my ultrasound training session	ST	0.333	0.517
	UI	0	1

## Discussion

Our pilot study showed high accuracy in identifying all volar arm structures using ultrasound in both groups, with the highest being the identification of radial and ulnar arteries. The ulnar nerve was the least correctly identified structure in the UI group, and the radial nerve was the least correctly identified structure in the ST group. Our findings are consistent with previous studies where nerve identification was best performed in a survey pattern [11]. The lack of proper surveying pattern and characteristic “honeycomb” mixed visual opacity of nerves could have made it more difficult for students to identify the nerves in both the UI and ST groups, while the generally anechoic appearance of blood vessels could have been easier for MSK ultrasound-naive students to identify [12]. There was no difference in confidence levels or identification accuracy when identifying volar arm structures between the UI and ST groups. This is consistent with our previous study that students were confident in identifying structures when taught by faculty or peers [13]. These results suggest that the effectiveness of ultrasound training in identifying the volar arm structures was relatively similar between STs and UIs. Other studies have supported that STs can provide sufficient ultrasound training in comparison to ultrasound faculty using an ST-led ultrasound curriculum [14,15].

UIs scored higher in teaching basic ultrasound concepts, suggesting that the general ultrasound knowledge and experience UIs have might provide a stronger foundational learning experience for students learning ultrasound than they would receive from STs. Also, greater teaching experience of UIs likely increased their awareness of the importance of providing a general ultrasound education first, whereas STs likely started teaching MSK-related ultrasound immediately without introducing the basics of ultrasound at the beginning of the session. Additionally, there was no statistically significant correlation between confidence and accuracy for any individual structure in both groups. This lack of correlation can be explained by having limited time to practice MSK ultrasound. Increased exposure time with ultrasound has been linked with a correlated increase in confidence and accuracy [16], but students were limited to one-hour workshops in this study. However, student confidence correlated (γ = 0.778) with overall identification accuracy in the ST group, while there was no correlation between overall student confidence and accuracy in the UI group. Due to the scarcity of UIs, a higher number of students were assigned to the UI group per session than the ST group. The decrease in individualized attention and further decreased ultrasound probe practice time in the UI group versus the ST group could have affected the confidence of the UI group students, despite their overall accuracy in identifying structures.

A limitation of this study was the small number of participants, which may hide statistically significant correlations between student confidence and accuracy in identifying ultrasound MSK structures. Another limitation of the study was the unequal number of UIs compared to STs when conducting the ultrasound sessions. Although we divided the session into multiple times to mitigate the difference in the number of students per UI/ST, having only two UIs led to an increased number of students in their ultrasound sessions compared to ST sessions. Despite this, students taught by UIs scored highly on their performances. Students taught by STs scored highly as well and had the slight advantage of fewer students per tutor. This could indicate that new STs would benefit from having a smaller group to teach in the beginning while slowly increasing the number of students they teach as they gain more experience.

The support of STs in teaching even difficult organ systems, such as MSK ultrasound, has encouraged us to expand the student-led ultrasound workshops we have held over the past year. We hope to not only continue the current ultrasound curriculum but to broaden the scope to include a student-led preclinical ultrasound scholar track. A preclinical ultrasound scholar program has been implemented at another medical school with relatively high satisfaction rates from STs [17]. Programs such as the ultrasound scholar program can be a self-sufficient and effective way to motivate student interest and increase student experience with ultrasound. Ultrasound exposure in preclinical years is correlated with increased understanding of anatomy and physiology, student ability and confidence in performing physical examinations, and opportunities for teaching and research [18,19].

## Conclusions

Ultrasound education in medical schools builds students’ medical knowledge, clinical skills, and leadership ability. STs are a cost-effective way to disperse MSK ultrasound knowledge in low-resource areas while providing STs with valuable leadership skills at the same time. Although previous literature supports the effectiveness of student-led ultrasound teaching in general, our pilot study supports the viability of using STs to teach MSK ultrasound specifically, despite the increase in difficulty compared to other organ systems. Further studies that include a larger sample size are needed to better understand the long-term benefits and possible logistical challenges in implementing MSK ultrasound using student instructors. Overall, MSK ultrasound’s high clinical utility and skill-based execution are valuable motivating factors for students to master complex ultrasound in their preclinical years while broadening their scope of clinical experiences.
